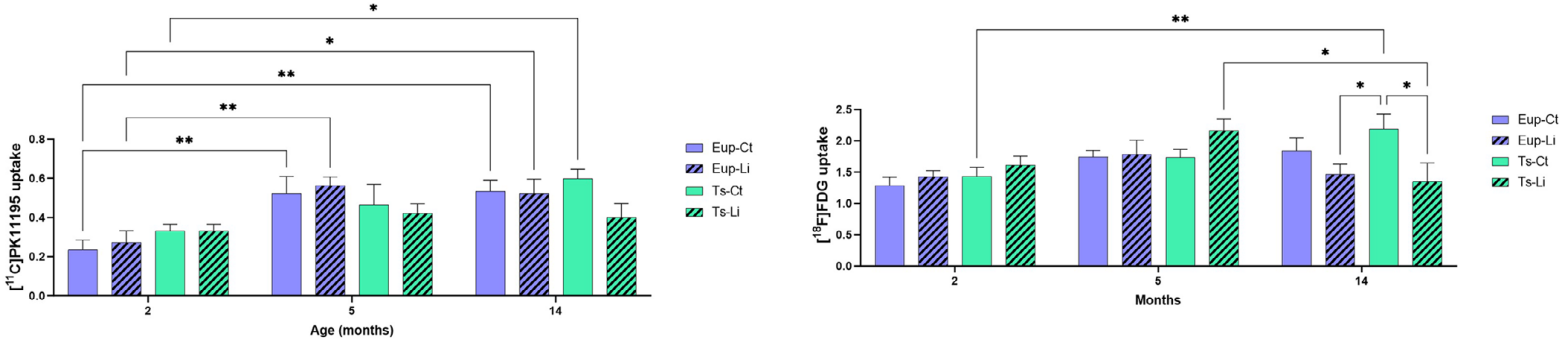# Evaluation of lithium carbonate treatment in a mouse model of Down syndrome using positron emission tomography

**DOI:** 10.1002/alz70856_099817

**Published:** 2025-12-24

**Authors:** Carolina Aparecida de Faria Almeida, Larissa Estessi de Souza, Chiara Maria Righini, Jean Marques Brizola, Carlos Alberto Buchpiguel, Lidia Emmanuela Wiazowski Spelta, Daniele de Paula Faria

**Affiliations:** ^1^ University of São Paulo Medical School, São Paulo, São Paulo, Brazil; ^2^ Universidade de São Paulo, São Paulo, SP, Brazil

## Abstract

**Background:**

Alzheimer's Disease (AD) is highly prevalent in people with Down syndrome (DS) due to the trisomy 21. As the life expectancy of people with DS has increased, new therapy strategies to delay the development of AD became an important area of research. Lithium carbonate (Li) treatment has showed neuroprotective effects in pre‐clinical studies, suggesting its potential as an intervention. Thus, this study aims to evaluate the effect of chronic Li treatment in a mouse model of DS, using positron emission tomography (PET).

**Method:**

Euploid (Eup) and trisomic (Ts) Ts65Dn mice (ethics approval: 1292/2019) were treated with Li (0.25 mg/kg) from 2 to 14 month‐old. Controls (Ct) received no treatment. [^11^C]PK11195 and [^18^F]FDG PET were performed to access neuroinflammation and brain metabolism at 2, 5 and 14 months‐old.

**Result:**

In Eup‐Ct and Eup‐Li, [^11^C]PK11195 uptake increased from 2 to 5 months (*p* <0.01 for both) and from 2 to 14 months (*p* <0.01 and *p* <0.05, respectively). In Ts‐Ct, [^11^C]PK11195 increased at 14 months (*p* <0.05) compared to 2 months, but in the Ts‐Li group the treatment avoided this alteration. Regarding [^18^F]FDG, its uptake also increased in Ts‐Ct from 2 to 14 months (*p* <0.001) and was higher than in Eup‐Li and Ts‐Li (*p* <0.05). Ts‐Li showed decreased [^18^F]FDG uptake from 5 to 14 months (*p* <0.05).

**Conclusion:**

Trisomic animals had an increase of both PET tracers uptake at 14 months, indicating the presence of neuroinflammation. Chronic lithium treatment prevented these alterations suggesting an anti‐neuroinflammatory effect that might be better comprehended in future studies.